# LNMGAT: a laplacian regularized pseudo-negative mining graph attention network for robust drug–target interaction prediction under multi-scenario cold-start settings

**DOI:** 10.3389/fbinf.2026.1882476

**Published:** 2026-07-31

**Authors:** Shuai Guo, Weichi Liu, Jie Zou, Tao Ban, Gaifang Dong

**Affiliations:** 1 College of Computer and Information Engineering, Inner Mongolia Agricultural University, Hohhot, China; 2 Inner Mongolia Autonomous Region Key Laboratory of Big Data Research and Application of Agriculture and Animal Husbandry, Hohhot, China; 3 College of Food Science and Engineering, Inner Mongolia Agricultural University, Hohhot, China; 4 Key Laboratory of Dairy Biotechnology and Engineering, Ministry of Education, Inner Mongolia Agricultural University, Hohhot, China

**Keywords:** cold-start generalization, drug–target interaction prediction, graph attention network, laplacian regularized least squares, reliable negative mining, true-negative-free learning

## Abstract

Computational drug–target interaction (DTI) prediction provides a scalable alternative to costly and time-consuming experimental screening, but its reliability is limited by the scarcity of experimentally verified negative interactions. In public DTI databases, most unobserved drug–target pairs are unlabeled rather than true non-interactions. Randomly treating these unlabeled pairs as negatives can introduce label noise and reduce model reliability, particularly in cold-start scenarios involving unseen drugs or targets. To address this issue, we propose LNMGAT, a LapRLS-guided reliable pseudo-negative mining framework coupled with dual graph attention encoders. Instead of relying on experimentally confirmed negative labels or randomly sampled negatives, LNMGAT first applies Laplacian regularized least squares to drug and target similarity graphs to identify low-confidence unlabeled pairs as reliable pseudo-negatives. Drug and target representations are then learned separately on similarity-based k-nearest-neighbor graphs using graph attention networks, and their embeddings are concatenated for MLP-based interaction prediction. Across Yamanishi, Davis, KIBA, and BindingDB benchmarks, LNMGAT achieved the best AUPR in 10 of 16 evaluation settings and ranked within the top two in 14 of 16 settings. In the 12 cold-start settings, LNMGAT obtained the best AUPR in 9 cases, with absolute AUPR gains over the strongest baseline of up to 0.016 in pair cold-start prediction. External evaluation on DrugBank positive interactions and SwissDock-based molecular docking further provided database-level and *in silico* support for the plausibility of high-ranked predictions. Nevertheless, the biological validation in this study remains computational and database-based; no wet-lab binding assay was performed. LNMGAT therefore provides a competitive and interpretable framework for DTI prediction under negative-label uncertainty, while further experimental validation is required for its top-ranked candidates.

## 1 Introduction

The development of drug–target interaction (DTI) prediction has evolved from early statistical models to modern deep learning frameworks, driven primarily by the growing demand for scalable and cost-effective drug discovery methods. Traditional drug development heavily relies on experimental screening to identify interactions between compounds and protein targets, a process that is both time-consuming and expensive, thereby motivating computational approaches. Early methods largely depended on handcrafted features, such as amino acid composition, position-specific scoring matrices, and molecular fingerprints, combined with classifiers like rotation forests and ensemble models, as well as feature selection strategies like IWSSR to reduce noise and redundancy in high-dimensional data (Suruliandi et al., 2024; Abbasi Mesrabadi et al., 2023; Taheri et al., 2024). Although these approaches laid the foundation for data-driven DTI modeling and performed well on benchmark datasets, their generalization to novel molecular structures or unseen target classes was limited, highlighting the need for more expressive representations and adaptive learning mechanisms.

To address these limitations, graph-based learning methods and advanced representation models have become central to modern DTI prediction. Graph neural networks (GNNs) are particularly suitable for molecular data, as they can model non-Euclidean structures. For instance, GraphormerDTI integrates structural information such as node spatial encoding and edge features via a graph Transformer encoder while combining CNNs for protein sequence processing (Gao et al., 2024); GSL-DTI dynamically learns optimal graph structures to enhance drug–target complex representations, improving predictive accuracy (Zixuan et al., 2024); GENNDTI introduces biologically inspired routing nodes to facilitate information propagation within graphs, producing structurally rich and biologically interpretable predictions (Yang et al., 2024a). Multimodal frameworks further enhance performance by integrating diverse information sources, e.g., MGNDTI uses gated mechanisms to fuse sequence- and graph-based embeddings, whereas TripletMultiDTI employs a triplet loss to improve separability of drug–target pairs (Peng et al., 2024; Dehghan et al., 2023); NFSA-DTI leverages self-attention to emphasize relevant molecular and protein substructures, enhancing the contextual depth of learned embeddings (Liu et al., 2024a).

Meanwhile, Transformer-based architectures have reshaped DTI prediction by capturing long-range dependencies and contextual representations. CAT-DTI introduces a cross-attention mechanism to model interactions between drug and protein representations, with domain adaptation to mitigate distribution shifts (Zeng et al., 2024); Multi-TransDTI employs universal dictionary encoding and multi-view Transformers to efficiently extract residue-level features, reducing sequence dimensionality (Wang et al., 2022); FastDTI integrates pre-trained Transformers with graph networks in a self-supervised learning pipeline for accelerated inference (Boezer et al., 2023). Given the black-box nature of deep learning models, interpretability has become a key concern. AttentionSiteDTI draws inspiration from NLP sentence classification to identify binding site contributions (Yazdani-Jahromi et al., 2022); DeFuseDTI enhances transparency using dual-branch encoders and multi-view attention fusion modules (Feng et al., 2024a); CSDTI and UdanDTI further advance interpretability and debiasing, with UdanDTI specifically mitigating common drug bias in conventional deep models (Pan et al., 2023; Zhang et al., 2025).

Self-supervised and semi-supervised learning strategies have also garnered attention for handling limited labeled data. SSLDTI applies self-supervised training to graph autoencoders with multi-level representations and imbalance compensation (Liu et al., 2024b); SHGCL-DTI leverages heterogeneous graph contrastive learning to improve embedding quality (Yao et al., 2023); PSF-DTI incorporates a pseudo-labeling framework within graph fusion attention networks to enhance reliability under sparse annotations (Xie et al., 2025). Heterogeneous networks facilitate richer relational modeling: NGCN constructs topologically diverse networks from biological data sources and applies graph convolutions (Cao et al., 2024); DMHGNN uses a dual multi-view architecture to capture perspectives from heterogeneous graphs and similarity matrices (Ning et al., 2025); DTI-HAN combines semantic-level bidirectional attention with an improved loss function to reduce label noise (Yu et al., 2024); ADEM further innovates by automatically learning effective meta-paths under similarity constraints (Zhang et al., 2024a).

Recent studies increasingly focus on higher-order structures and global topological signals. CHL-DTI integrates binary interaction matrices with heterogeneous semantic associations, leveraging hypergraphs to capture richer structural context (Wang et al., 2024); FBRWPC implements a fine-grained filtering process using bidirectional random walks and probabilistic similarity selection to enhance graph quality (Wang and Yin, 2024); AMGDTI employs flexible metagraph approaches to automatically learn latent semantic paths without predefined patterns (Su et al., 2023). Hybrid architectures that fuse multiple neural modules and feature domains have also emerged. SiamDTI uses a dual-channel strategy to integrate local and global protein context, improving zero-shot performance (Zhang et al., 2024b); DrugSchizoNet optimizes an end-to-end workflow for schizophrenia-targeted drug discovery (Sherine Glory et al., 2025); GCARDTI combines SELFIES (a robust SMILES alternative) with CNN-GAT hybrid models to improve drug encoding and interaction detection (Feng et al., 2024b).

Integrating domain knowledge with scalable modeling approaches has further advanced the field. Techniques such as interpretable feature selection pipelines and autoencoder-based weight allocation effectively reduce feature redundancy and enhance transparency (Kotkondawar et al., 2025; Liao et al., 2025). The rise of large language models (LLMs) is beginning to impact DTI tasks: DrugAgent introduces a multi-agent LLM reasoning framework, incorporating literature- and structure-based evidence into predictions (Inoue et al., 2025), complemented by reviews outlining LLM potential and limitations in drug discovery (AbuNasser et al., 2024). Cold-start prediction—where drugs or targets lack interaction data—has also been addressed: MGDTI employs meta-learning with similarity metrics to generalize to new entities (He et al., 2025); MatchMaker extends this concept to the proteome level, integrating chemoinformatics and docking to identify ligands for challenging targets such as DCAF1 (Kimani et al., 2023). Continuous-valued affinity modeling has similarly gained traction: MT-DTA uses cross-attention and variational autoencoders to estimate binding strength and quantify uncertainty (Zhu et al., 2023), while PGraphDTA leverages protein contact graphs and pre-trained embeddings to enhance structural resolution in affinity prediction (Bal et al., 2023).

Scalability and model efficiency remain critical for practical deployment. BarlowDTI uses a Barlow Twins contrastive framework to disentangle embeddings and lightweight gradient boosting for final classification (Schuh et al., 2025); studies on embedding aggregation strategies highlight the importance of architecture choices in multi-branch DTI frameworks (Embedding Aggregation Study, 2024); SMOTE and random undersampling improve prediction stability in imbalanced datasets (Azlim Khan and Malim, 2023); FDTIIT minimizes feature redundancy via cross-attention (He et al., 2024); DeepMGT-DTI and DeepPS integrate graph, Transformer, and convolutional layers to enable high-throughput inference with minimal accuracy loss (Zhang et al., 2024c; D’Souza et al., 2023); metapath-guided frameworks such as BG-DTI and semantic-aware path learning provide effective solutions for sparse and incomplete datasets (Molaee et al., 2025; Li et al., 2024). Additional strategies—including KL-divergence-based random forests, capsule networks with transfer learning, and adversarial learning for multimodal domain generalization—demonstrate the growing diversity and creativity in addressing DTI prediction challenges (Ahn et al., 2024; Huang et al., 2023; Hua et al., 2025).

Although recent DTI prediction studies have introduced increasingly expressive graph-based, Transformer-based, and multimodal learning frameworks, the reliability of model supervision remains a central concern. In public DTI databases, experimentally verified negative interactions are rarely available, and most unobserved drug-target pairs are unlabeled rather than confirmed non-interactions. Directly treating these unlabeled pairs as negatives may introduce false-negative labels and distort the learned decision boundary. This problem becomes more pronounced in cold-start settings, where newly developed drugs, newly characterized targets, or entirely novel drug-target pairs have limited or no interaction evidence. Therefore, a practical DTI prediction framework should not only learn informative representations, but also reduce the uncertainty introduced by unreliable negative supervision.

To address these issues, we propose LNMGAT, a LapRLS-guided reliable pseudo-negative mining graph attention network for DTI prediction under negative-label uncertainty and multi-scenario cold-start settings. The main advantage of LNMGAT lies in its integration of two complementary components. First, LapRLS is used to propagate similarity-based information over drug and target graphs and to identify low-confidence unlabeled pairs as reliable pseudo-negative samples, reducing the dependence on random negative sampling. Second, dual GAT encoders learn drug and target representations from similarity-based k-nearest-neighbor graphs, enabling the model to capture local neighborhood structures and higher-order similarity patterns before MLP-based interaction prediction. Meanwhile, we acknowledge that the mined pseudo-negative samples are not experimentally verified non-interactions, and that the cold-start settings are evaluated with fixed similarity priors rather than in a fully inductive setting. Thus, LNMGAT is designed as a practical and interpretable framework for improving DTI prediction reliability under sparse annotations, uncertain negative labels, and cold-start generalization challenges.

## 2 Materials and methods

### 2.1 Datasets and preprocessing

We employed four benchmark datasets commonly used in the drug–target interaction (DTI) prediction field, including the Yamanishi, Davis, KIBA, and BindingDB datasets (the latter using the dissociation constant Kd as labels). Table 1 summarizes the key statistics of these datasets, such as the numbers of drugs and targets, the total number of drug–target pairs, and the number of interacting pairs. The Yamanishi dataset consists of four subsets: Yamanishi-Enzyme (Y-E), Yamanishi-Ion Channel (Y-C), Yamanishi-GPCR (Y-G), and Yamanishi-Nuclear Receptor (Y-N). It is worth noting that the original Davis, KIBA, and BindingDB datasets are not inherently binary classification datasets. In this study, we follow commonly adopted practices in the literature and convert them into binary datasets using affinity thresholds: 7.0 for Davis and BindingDB, and 12.1 for KIBA (Zhan et al., 2025; Luo et al., 2024; Chen et al., 2024).

**TABLE 1:** Datasets overview.

Dataset	Drug/target	Total pair	Interaction
Yamanishi- enzyme (Y-E)	445/664	295,480	2,926
Yamanishi- ion channel (Y-C)	210/204	42,840	1,476
Yamanishi- GPCR (Y-G)	223/95	21,185	635
Yamanishi- nuclear receptor (Y-N)	54/26	1,404	60
Davis	68/379	25,772	1,784
KIBA	2,069/229	117,657	24,675
BindingDB	34,366/2,296	82,037	26,533

BindingDB database contains various types of affinity labels, including dissociation constants ( $K_{d}$ ), inhibition constants ( $K_{i}$ ), and half-maximal inhibitory concentrations ( $IC_{50}$ ). For consistency and rigor in subsequent modeling, this study exclusively utilized the dissociation constant ( $K_{d}$ ) values to represent the binding affinity.

The raw $K_{d}$ values, typically reported in nanomolar ( nM ) units, were parsed to obtain a uniform numerical measure. Since the raw data often contains textual elements like inequality signs ( >,<,≤,≥ ), these were resolved by setting the numerical threshold as the $K_{d}$ value in nM . Data points that could not be reliably parsed or possessed non-positive $K_{d}$ values were filtered out.

To establish a continuous, positive, and logarithmically scaled endpoint suitable for quantitative analysis, the numerical $K_{d}$ values were transformed into the $pK_{d}$ metric. This transformation first requires converting the $K_{d}$ value from nM to molar ( M ) units, which is achieved by multiplying the nM value by $10^{-9}$ . As shown in Equation 1, The $pK_{d}$ value is then calculated using the negative base-10 logarithm of the molar concentration ( $K_{d,M}$ ):

$$pK_{d}=-\log_{10}\left(K_{d,M}\right)$$ (1)

The final formula used for the conversion is given by Equation 2:

$$pK_{d}=-\log_{10}\left(K_{d,\text{nM}}\times 10^{-9}\right)$$ (2)

This conversion ensures that a higher $pK_{d}$ value directly corresponds to a stronger binding affinity.

### 2.2 Overview of LNMGAT DTI prediction pipeline construction

**FIGURE 1:**
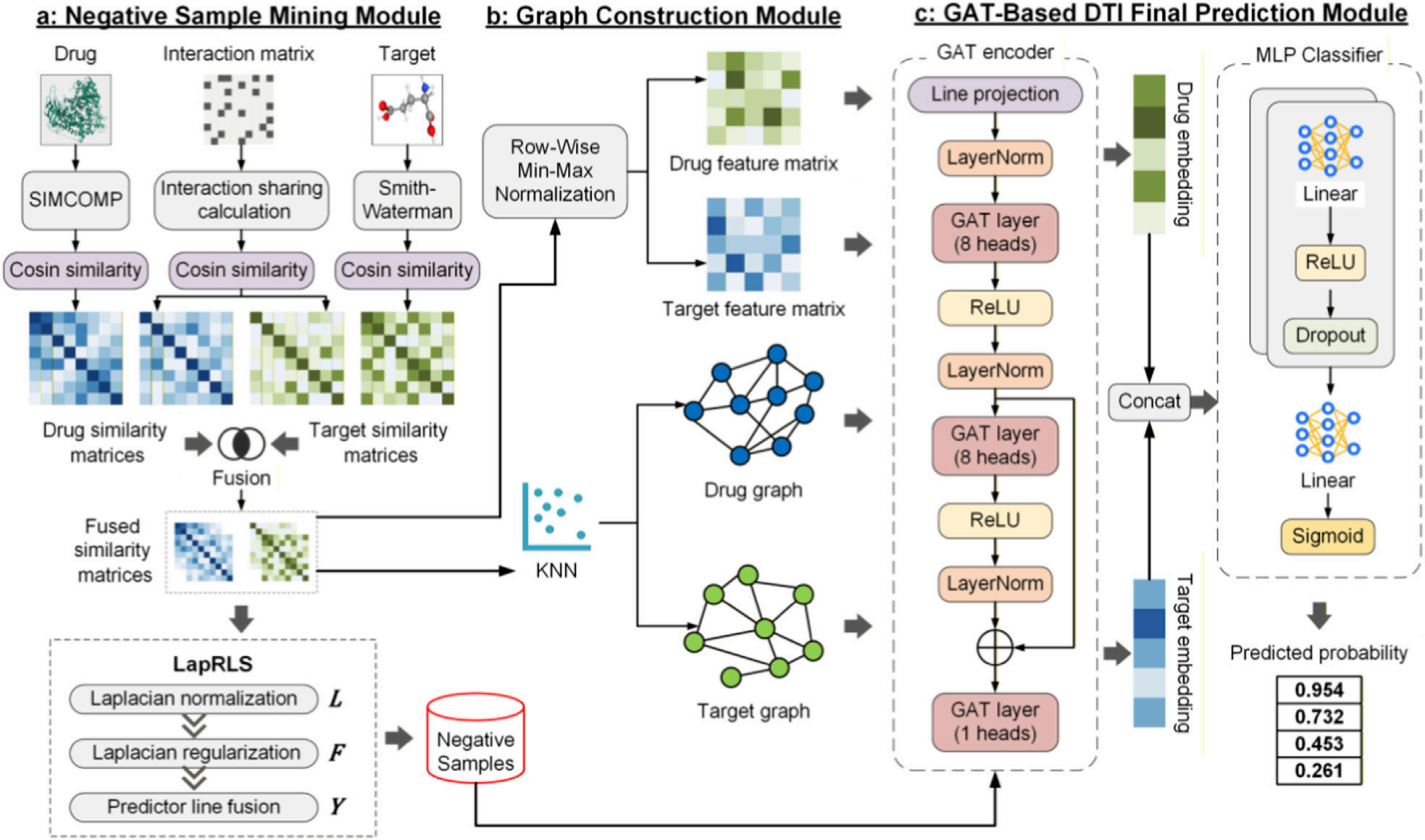
Framework of LNMGAT prediction pipeline. **(a)** Negative sample mining module. **(b)** Graph construction module. **(c)** GAT-based DTI final prediction module.

As shown in Figure 1, the proposed model LNMGAT (LapRLS-Driven Negative Mining Graph Attention Network) consists of three hierarchical modules processing information from left to right: (a) LapRLS-Based Negative Sample Mining Module: Multiple drug and target similarity matrices are fused and normalized. A Laplacian Regularized Least Squares (LapRLS) predictor utilizes graph smoothness constraints to estimate interaction scores for unlabelled pairs. Pairs with the lowest scores are selected as “Reliable Negative Samples” to address the class imbalance problem in training data. (b) Graph Construction Module: The unified similarity representations are processed via row-wise Min-Max normalization to generate initial Feature Matrices. Simultaneously, structural information is encoded by constructing Drug/Target k-Nearest Neighbor (k-NN) Graphs, where edges are defined based on similarity rankings. (c) GAT-Based Prediction Module: The Drug GAT Encoder and Target GAT Encoder extract latent high-order features using three stacked Graph Attention layers (incorporating multi-head attention and residual connections). Finally, the learned drug and target embeddings are concatenated and fed into an MLP Classifier to output the final predicted probability of interaction.

### 2.3 Negative sample mining

In this study, we distinguish three types of negative samples in drug–target interaction prediction. Experimentally verified negative interactions refer to drug–target pairs confirmed not to interact in wet-lab experiments, which are rarely available in public datasets. Random negatives are unlabeled pairs randomly selected and assumed to be non-interactions, which may introduce label noise due to hidden positive interactions. In contrast, reliable pseudo-negative samples refer to unlabeled pairs with low interaction scores estimated by the LapRLS model, which are used as negative supervision during training. Therefore, LNMGAT does not rely on experimentally verified negative labels or random negative sampling, but instead constructs pseudo-negative supervision from unlabeled data.

#### 2.3.1 Interaction and similarity matrices calculation

The interaction matrix is defined by Equation 3:

$$Y\in R^{n\times m},Y_{ij}=\left\{\begin{array}{ll} 1, & \text{if drug }d_{i}\text{ interacts with target }t_{j}\\ 0, & \text{otherwise} \end{array}\right.$$ (3)

where n and m denote the number of drugs and targets, respectively. It is particularly important to note that entries with $Y_{ij}=0$ may represent unknown interactions, which could include novel drug–target pairs not yet discovered.

In addition, the Yamanishi dataset provides drug–drug similarity matrices ( $S_{d}^{struct}$ ) and target–target similarity matrices ( $S_{t}^{seq}$ ), computed using the SIMCOMP algorithm (based on chemical structures) and the Smith–Waterman algorithm (based on protein sequences). This multi-source similarity information supplies a rich foundation for downstream modeling. For the Davis, KIBA and BindingDB datasets, since no precomputed drug and target similarity matrices are available, we constructed them separately by calculating drug similarities based on their SMILES using the SIMCOMP algorithm, and target similarities based on their sequences using the Smith–Waterman method.

#### 2.3.2 Interaction sharing similarity matrix calculation

We first construct the interaction sharing matrix. Taking drugs as an example: if drug $d_{1}$ and drug $d_{2}$ share two common targets, the entry is set to 2; if they share none, the value is 0. All diagonal elements are set to 1. The interaction sharing similarity matrix is then calculated using cosine similarity (Equation 4):

$$S_{d}^{\text{share}}\left(i,j\right)=\frac{M_{i}\cdot M_{j}}{\left\|M_{i}\right\|\left\|M_{j}\right\|},S_{t}^{\text{share}}\left(i,j\right)=\frac{N_{i}\cdot N_{j}}{\left\|N_{i}\right\|\left\|N_{j}\right\|}$$ (4)

This step captures functional associations between entities by quantifying shared interactions, thus providing additional biological relevance.

#### 2.3.3 Similarity fusion

To integrate multiple similarity sources, we adopt a linear fusion strategy, yielding the final drug similarity matrix $S_{d}$ and target similarity matrix $S_{t}$ . Assuming equal contributions from structural/sequence similarity and interaction sharing similarity, the fusion is defined by Equation 5:

$$S_{d}=\alpha S_{d}^{struct}+\left(1-\alpha \right)S_{d}^{share},S_{t}=\alpha S_{t}^{seq}+\left(1-\alpha \right)S_{t}^{share}$$ (5)

This strategy ensures robust and comprehensive similarity representations by leveraging complementary modalities.

#### 2.3.4 Laplacian normalization and regularization

We normalize the similarity matrices by computing their corresponding degree matrices $D_{d}$ and $D_{t}$ . For a similarity matrix S , the degree matrix is defined as a diagonal matrix where each diagonal element equals the sum of the corresponding row (Equation 6):

$$D_{d}\left(i,i\right)=\sum_{j}\;S_{d}\left(i,j\right),D_{t}\left(i,i\right)=\sum_{j}\;S_{t}\left(i,j\right)$$ (6)

This definition ensures that $D_{d}$ and $D_{t}$ reflect the overall connectivity (or influence) of each drug and target within the similarity graph.

Using these, the normalized similarity matrices are computed by Equation 7:

$${\tilde{S}}_{d}=D_{d}^{-\frac{1}{2}}S_{d}D_{d}^{-\frac{1}{2}},{\tilde{S}}_{t}=D_{t}^{-\frac{1}{2}}S_{t}D_{t}^{-\frac{1}{2}}$$ (7)

The Laplacians are then defined by Equation 8:

$$L_{d}=D_{d}-{\tilde{S}}_{d},L_{t}=D_{t}-{\tilde{S}}_{t}$$ (8)

The LapRLS prediction functions are defined by Equation 9:

$$F_{d}={\left(K_{d}+\lambda_{d}L_{d}\right)}^{-1}Y,F_{t}=Y{\left(K_{t}+\lambda_{t}L_{t}\right)}^{-1}$$ (9)

where $K_{d}$ and $K_{t}$ are the kernel matrices for drugs and targets, and $\lambda_{d},\lambda_{t}$ are hyperparameters controlling the regularization strength.

Finally, the prediction matrix is obtained using Equation 10:

$$\hat{Y}=\frac{F_{d}+F_{t}}{2}$$ (10)

From $\hat{Y}$ , known positive interactions are retained from the training fold, while the lowest-scoring unlabeled pairs are selected as reliable pseudo-negative samples. These pseudo-negative samples are not experimentally verified non-interactions, but low-confidence unlabeled pairs identified by LapRLS, thereby constructing a high-confidence labeled dataset for downstream representation learning.

### 2.4 Graph construction for heterogeneous information encoding

The foundation of our drug-target interaction (DTI) prediction model lies in constructing a heterogeneous information network (HIN) that seamlessly integrates multi-view similarity structures and known interaction data. This module refines the input matrices $S_{d}$ and $S_{t}$ and formalizes the graph structure for subsequent learning.

#### 2.4.1 Node definition and feature initialization

The constructed heterogeneous graph $G=\left(V,E\right)$ comprises a set of nodes $V=V_{d}\cup V_{t}$ and a set of edges E .

##### 2.4.1.1 Node sets

The disjoint node sets represent drugs and targets are given by Equations 11, 12:

$$V_{d}=d_{1},d_{2},\ldots ,d_{n}$$ (11)

$$V_{t}=t_{1},t_{2},\ldots ,t_{m}$$ (12)

where n and m are the total number of drugs and targets, respectively.

##### 2.4.1.2 Feature initialization

We leverage the refined similarity matrices to initialize the raw feature vector $x_{v}$ for each node v∈V . As shown in Equations 13, 14, this approach ensures that the initial node embeddings inherently capture global structural and sequential characteristics:

$$x_{d_{i}}=S_{d}\left(i,:\right)\in R^{n}$$ (13)

$$x_{t_{j}}=S_{t}\left(j,:\right)\in R^{m}$$ (14)

This initialization strategy effectively transforms the entity similarity information into a dense, high-dimensional feature representation $X\in R^{\left(n+m\right)\times \max\left(n,m\right)}$ .

#### 2.4.2 Edge definition and graph topology

The edge set E is partitioned into three distinct types, enabling the GAT model to concurrently learn from functional associations and intrinsic homologies.

##### 2.4.2.1 Cross-type interaction edges

These edges represent the ground-truth interactions between drug and target entities, including the high-confidence negative samples $Y_{neg}$ identified by the mining strategy. The set is formally defined by Equation 15:

$$E_{dt}=\{\left(d_{i},t_{j}\right)|Y_{ij}\left.\in \{0,1\}\right\}$$ (15)

Here, $Y_{ij}=1$ denotes a known positive interaction, and $Y_{ij}=0$ denotes a pseudo-negative label assigned to a low-confidence unlabeled pair for supervised training, rather than an experimentally verified non-interaction. This set forms the crucial structural link for DTI prediction.

##### 2.4.2.2 Within-type similarity edges

Edges within the same entity type propagate latent contextual information derived from the fused similarity measures. These edges are typically weighted.

Drug-Drug Edges (Equation 16):

$$E_{dd}=\left\{\left(d_{i},d_{j},w_{ij}\right)|S_{d}\left(i,j\right)>\tau_{d}\right\}$$ (16)

Target-Target Edges (Equation 17):

$$E_{tt}=\left\{\left(t_{i},t_{j},w_{ij}\right)|S_{t}\left(i,j\right)>\tau_{t}\right\}$$ (17)

where $w_{ij}$ is the weight $S_{d}\left(i,j\right)$ or $S_{t}\left(i,j\right)$ , and $\tau_{d}$ and $\tau_{t}$ are pre-defined similarity thresholds used to prune weak, unreliable edges and reduce graph sparsity.

### 2.5 GAT-based representation learning and prediction

The normalized heterogeneous graph is then fed into a multi-layer Graph Attention Network (GAT) to learn task-specific, low-dimensional latent representations for all entities. The attention mechanism is crucial for differentially weighing the contributions of heterogeneous neighbors (similar drugs, similar targets, and interacting partners).

#### 2.5.1 Multi-head attention mechanism

The GAT layer transforms the input features $H=\left\{h_{1},\ldots ,h_{n+m}\right\}$ into output features $H^{\prime }=\left\{h_{1}^{\prime },\ldots ,h_{n+m}^{\prime }\right\}$ . For a node v and its neighbor u , the attention coefficient $e_{vu}^{\left(k\right)}$ for the k -th attention head is calculated after a linear transformation $W^{\left(k\right)}$ and concatenation (Equation 18):

$$e_{vu}^{\left(k\right)}=\text{LeakyReLU}\left(a^{\left(k\right)⊤}\left[W^{\left(k\right)}h_{v}\|W^{\left(k\right)}h_{u}\right]\right)$$ (18)

where $W^{\left(k\right)}\in R^{F^{\prime }\times F}$ is the weight matrix, $a^{\left(k\right)}\in R^{2F^{\prime }}$ is the learnable attention vector, and $F^{\prime }$ is the output feature dimension of the head.

As shown in Equation 19, the resulting attention coefficients are then normalized across all neighbors $N\left(v\right)$ of node v using the softmax function to obtain the final attention weights $\alpha_{vu}^{\left(k\right)}$ :

$$\alpha_{vu}^{\left(k\right)}=\frac{\text{ex}p\left(e_{vu}^{\left(k\right)}\right)}{\sum_{u^{\prime }\in N\left(v\right)}\;\text{ex}p\left(e_{vu^{\prime }}^{\left(k\right)}\right)}$$ (19)

This attention weight $\alpha_{vu}^{\left(k\right)}$ quantifies the relative importance of neighbor u to node v within the context of the k -th attention head.

#### 2.5.2 Feature aggregation and update

The node’s new feature representation $h_{v}^{\prime }$ is computed by aggregating and linearly combining the features of its neighbors, weighted by the normalized attention coefficients. The use of K independent attention heads (multi-head attention) stabilizes the learning process and enhances expressive power.

The aggregation across all heads is typically done by concatenation (for non-output layers) using Equation 20:

$$h_{v}^{\prime }\left.=\right|{k=1}^{K}\sigma \left(\sum_{u\in N\left(v\right)}\;\alpha_{vu}^{\left(k\right)}W^{\left(k\right)}h_{u}\right)$$ (20)

where $\sigma \left(\cdot \right)$ is the Exponential Linear Unit (ELU) or Rectified Linear Unit (ReLU) activation function. After L layers of GAT, the final learned embeddings for drug $d_{i}$ and target $t_{j}$ are denoted as $z_{d_{i}}$ and $z_{t_{j}}$ .

#### 2.5.3 Drug–target interaction prediction

After the drug and target representations are learned by the two GAT encoders, LNMGAT uses an MLP classifier as the final prediction head. Specifically, for a candidate drug–target pair $\left(d_{i},t_{j}\right)$ , the learned drug embedding $h_{d_{i}}$ and target embedding $h_{t_{j}}$ are concatenated to form a pair-level representation (Equation 21):

$$z_{ij}=\left[h_{d_{i}}\|h_{t_{j}}\right]$$ (21)

where $h_{d_{i}}$ denotes the learned embedding of drug $d_{i}$ , $h_{t_{j}}$ denotes the learned embedding of target $t_{j}$ , and ‖ denotes vector concatenation.

The concatenated representation $z_{ij}$ is then fed into an MLP classifier consisting of fully connected layers, ReLU activation, and dropout regularization (Equations 22–24):

$$u_{ij}=\text{ReLU}\left(W_{1}z_{ij}+b_{1}\right)$$ (22)

$$v_{ij}=\text{ReLU}\left(W_{2}u_{ij}+b_{2}\right)$$ (23)

$$s_{ij}=W_{3}v_{ij}+b_{3}$$ (24)

The final interaction probability is obtained by applying the sigmoid function to the output logit (Equation 25):

$${\hat{y}}_{ij}=\sigma \left(s_{ij}\right)$$ (25)

where $W_{1}$ , $W_{2}$ , $W_{3}$ , $b_{1}$ , $b_{2}$ , and $b_{3}$ are trainable parameters of the MLP classifier. ${\hat{y}}_{ij}$ represents the predicted probability that drug $d_{i}$ interacts with target $t_{j}$ . This MLP-based prediction head replaces the previously described bilinear decoder and is consistent with Figure 1 and with the actual implementation, in which the learned drug and target embeddings are concatenated before classification.

#### 2.5.4 Model optimization and loss function

The model is trained end-to-end by minimizing the binary cross-entropy loss over known positive interactions and LapRLS-mined reliable pseudo-negative samples. For each training pair $\left(\left(d_{i},t_{j}\right)\right),$ the MLP classifier outputs the predicted interaction probability $\left({\hat{y}}_{ij}\right).$ The loss function is defined by Equation 26:

$$L_{BCE}=-\frac{1}{N}\sum_{\left(i,j\right)\in D}\left[y_{ij}\log\left(\hat{y}*ij\right)+\left(1-y*ij\right)\log\left(1-{\hat{y}}_{ij}\right)\right]$$ (26)

where (D) denotes the training set, and (N) is the number of training samples in (D). The label $\left(y_{ij}=1\right)$ denotes a known positive interaction, whereas $\left(y_{ij}=0\right)$ denotes a reliable pseudo-negative label assigned to a low-confidence unlabeled pair. The predicted probability $\left({\hat{y}}_{ij}\right)$ is generated by the MLP-based prediction head described in Section 2.5.3.

To reduce overfitting, dropout regularization and weight decay are applied during training. The model is optimized using the AdamW optimizer.

## 3 Experimental setup and performance evaluation

### 3.1 Implementation details and hyperparameter settings

The LNMGAT model is implemented using PyTorch and PyTorch Geometric. Key hyperparameters are summarized in Table 2. All experiments were conducted on a server running Ubuntu 22.04 with Python 3.12, PyTorch 2.8.0, and CUDA 12.8. The hardware platform was equipped with an Intel Xeon(R) Platinum 8458P CPU with 20 cores, 120 GB system memory, and one NVIDIA H800 GPU with 80 GB memory. A 50 GB data disk was used for dataset storage and experimental outputs.

**TABLE 2:** Key hyperparameter settings for the LNMGAT model.

Category	Hyperparameter	Value
Architecture	Hidden dimension	256
	Attention heads	8
	kNN neighbors	20
Optimization	Learning rate	0.0005
	Weight decay	0.0005
	Epochs	300
	Dropout rate	0.3
	Batch size	512
Sampling	Train pseudo-negative ratio	2:1
	Evaluation pseudo-negative ratio	3:1
	F1 threshold	0.5

### 3.2 Evaluation protocol and prediction scenarios

**FIGURE 2:**
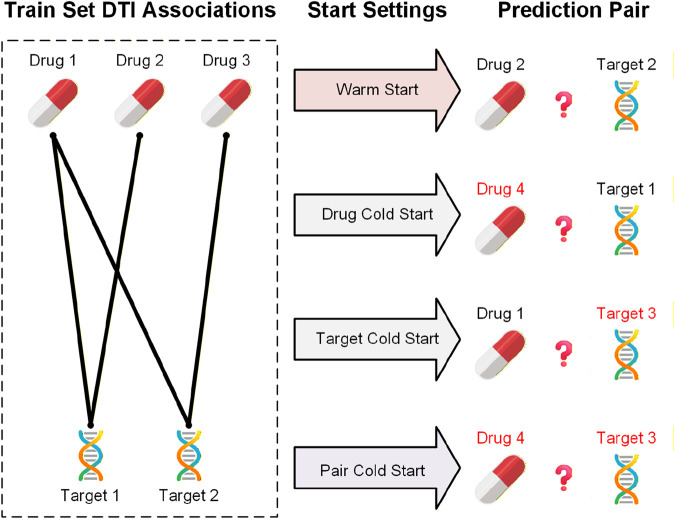
Illustration of warm-start and cold-start evaluation settings.

A stratified 5-fold cross-validation (5-CV) strategy is applied to the positive interaction set. In each fold, a strict masking procedure is enforced on the training matrix $A^{train}$ to simulate cold-start conditions (Figure 2). Performance is assessed across four distinct prediction scenarios detailed in Table 3, which categorize the generalization challenges based on whether the drug and target entities were observed in the training graph. Note that evaluation negatives are sampled from unlabeled pairs and should be interpreted as pseudo-negative samples rather than experimentally verified non-interactions.

**TABLE 3:** Four prediction scenarios for model evaluation.

Scenario name	Entity status in training graph	Test set composition (types of interactions)
Warm-start	Drug: Known Target: Known	Interactions between known drugs and known targets
Drug cold start	Drug: New Target: Known	Interactions involving drugs absent from the training set and known targets
Target cold start	Drug: Known Target: New	Interactions involving known drugs and targets absent from the training set.
Pair cold start	Drug: New Target: New	Interactions where neither the drug nor the target was observed in the training set.

Fold-wise masking and leakage control. To prevent direct leakage of test interaction labels into pseudo-negative mining, all LapRLS-based mining procedures were performed in a fold-wise manner. For each fold, a fold-specific training interaction matrix, denoted as A_train_k, was constructed before LapRLS inference. All interactions involving held-out drugs were masked by setting the corresponding drug rows to zero, all interactions involving held-out targets were masked by setting the corresponding target columns to zero, and warm-start test positive pairs were also removed from the training matrix. LapRLS was then applied to A_train_k, rather than to the full interaction matrix. Candidate pseudo-negative samples were restricted to unlabeled pairs within the training drug-by-target region and excluded all test positive pairs. Therefore, test interaction labels were not directly used during supervised training or LapRLS-based pseudo-negative selection.

The drug-drug and target-target similarity matrices were used as fixed side information for graph construction and LapRLS regularization. Accordingly, the cold-start settings in this study should be interpreted as entity-masked transductive evaluation with fixed similarity priors, rather than fully inductive evaluation without any prior information about test drugs or targets. This clarification was added to avoid overstatement of the leakage-control scope.

### 3.3 Evaluation metrics

The predictive performance of LNMGAT and all reproduced baseline methods was evaluated using five standard metrics. All results reported in Tables 4–7 are presented as mean values with standard deviations across the same 5-fold cross-validation procedure. The ranking performance is assessed via the Area Under the ROC Curve (AUROC), which quantifies the model’s overall discriminative capability, and the Area Under the Precision-Recall Curve (AUPR), which provides a robust measure of efficacy focused on the minority positive class.

**TABLE 4:** Performance comparison of different methods under multiple scenarios on the Yamanishi dataset.

Method	Warm start		Drug cold start		Target cold start		Pair cold start	
	AUPR	AUROC	AUPR	AUROC	AUPR	AUROC	AUPR	AUROC
MAARDTI	0.935 (0.012)	0.963 (0.008)	**0.556 (0.038)**	0.587 (0.029)	0.875 (0.021)	0.906 (0.014)	0.528 (0.051)	0.547 (0.037)
MGDTI	0.902 (0.015)	0.934 (0.011)	0.545 (0.041)	0.569 (0.032)	0.842 (0.025)	0.878 (0.017)	0.503 (0.048)	0.529 (0.034)
DrugLAMP	0.889 (0.018)	0.921 (0.013)	0.531 (0.036)	0.561 (0.030)	0.828 (0.022)	0.867 (0.016)	0.496 (0.044)	0.515 (0.031)
INDTI	**0.915 (0.014)**	**0.945 (0.010)**	0.553 (0.039)	0.575 (0.028)	0.853 (0.023)	0.888 (0.015)	0.512 (0.047)	0.538 (0.035)
MINDG	0.876 (0.020)	0.910 (0.015)	0.524 (0.040)	0.552 (0.033)	0.812 (0.027)	0.855 (0.018)	0.482 (0.050)	0.505 (0.038)
DTiGEMS+	0.903 (0.013)	0.938 (0.009)	0.549 (0.037)	0.566 (0.028)	0.846 (0.020)	0.882 (0.014)	0.509 (0.045)	0.533 (0.032)
NASNet-DTI	0.917 (0.011)	0.947 (0.007)	0.558 (0.035)	**0.578 (0.027)**	**0.858 (0.019)**	**0.891 (0.013)**	**0.518 (0.043)**	**0.541 (0.030)**
LNMGAT (ours)	0.912 (0.010)	0.941 (0.006)	0.565 (0.033)	0.603 (0.025)**	0.872 (0.018)	0.903 (0.012)	0.544 (0.040)*	0.566 (0.029)*

Color convention: best, 2nd-best, and **3rd-best**. Values are reported as mean with standard deviation in parentheses over 5-fold cross-validation. All baseline methods were reproduced in this study under the same preprocessing procedure, data partitions, cold-start scenario definitions, and pseudo-negative sampling protocol as LNMGAT., color convention indicates the best, second-best, and third-best values within each column. Statistical significance was assessed by comparing LNMGAT, with the strongest competing baseline in each setting using fold-level paired tests. * indicates p < 0.05, and ** indicates p < 0.01.

**TABLE 5:** Performance comparison of different methods under multiple scenarios on the Davis dataset.

Method	Warm start		Drug cold start		Target cold start		Pair cold start	
	AUPR	AUROC	AUPR	AUROC	AUPR	AUROC	AUPR	AUROC
MAARDTI	0.849 (0.008)	0.924 (0.006)	**0.622 (0.022)**	0.917 (0.015)	0.638 (0.019)	**0.927 (0.013)**	**0.620 (0.025)**	0.914 (0.018)
MGDTI	0.841 (0.009)	0.927 (0.007)	0.646 (0.021)	**0.931 (0.014)**	0.655 (0.017)	0.947 (0.012)	0.627 (0.022)	0.913 (0.016)
DrugLAMP	0.812 (0.010)	0.903 (0.009)	0.598 (0.024)	0.882 (0.018)	0.618 (0.020)	0.896 (0.015)	0.581 (0.026)	0.871 (0.019)
INDTI	**0.842 (0.008)**	0.919 (0.006)	**0.622 (0.020)**	0.904 (0.016)	0.637 (0.018)	0.913 (0.014)	0.601 (0.024)	0.889 (0.017)
MINDG	0.803 (0.011)	0.892 (0.010)	0.583 (0.027)	0.861 (0.020)	0.604 (0.022)	0.878 (0.017)	0.569 (0.029)	0.852 (0.021)
DTiGEMS+	0.838 (0.007)	0.913 (0.006)	0.618 (0.023)	0.897 (0.015)	0.631 (0.020)	0.909 (0.013)	0.596 (0.024)	0.886 (0.016)
NASNet-DTI	0.846 (0.008)	0.921 (0.006)	0.629 (0.021)	**0.921 (0.014)**	**0.643 (0.017)**	0.926 (0.011)	0.612 (0.023)	0.897 (0.015)
LNMGAT (ours)	0.841 (0.006)	**0.922 (0.004)**	0.648 (0.019)	0.935 (0.012)	0.662 (0.015)*	0.948 (0.010)	0.624 (0.018)	**0.908 (0.013)**

Color convention: best, 2nd-best, and **3rd-best**. Values are reported as mean with standard deviation in parentheses over 5-fold cross-validation. All baseline methods were reproduced in this study under the same preprocessing procedure, data partitions, cold-start scenario definitions, and pseudo-negative sampling protocol as LNMGAT., color convention indicates the best, second-best, and third-best values within each column. Statistical significance was assessed by comparing LNMGAT, with the strongest competing baseline in each setting using fold-level paired tests. * indicates p < 0.05, and ** indicates p < 0.01.

**TABLE 6:** Performance comparison of different methods under multiple scenarios on the KIBA dataset.

Method	Warm start		Drug cold start		Target cold start		Pair cold start	
	AUPR	AUROC	AUPR	AUROC	AUPR	AUROC	AUPR	AUROC
MAARDTI	**0.866 (0.010)**	0.947 (0.007)	0.642 (0.018)	0.914 (0.014)	0.634 (0.020)	0.901 (0.015)	0.613 (0.021)	0.895 (0.016)
MGDTI	0.864 (0.009)	0.952 (0.006)	0.657 (0.017)	**0.945 (0.012)**	**0.643 (0.018)**	**0.927 (0.013)**	0.647 (0.019)	**0.931 (0.014)**
DrugLAMP	0.831 (0.011)	0.939 (0.008)	0.628 (0.019)	0.914 (0.015)	0.612 (0.020)	0.897 (0.014)	0.598 (0.022)	0.881 (0.017)
INDTI	0.847 (0.010)	0.946 (0.007)	0.639 (0.018)	0.932 (0.013)	0.622 (0.019)	0.912 (0.014)	0.609 (0.020)	0.901 (0.015)
MINDG	0.802 (0.012)	0.918 (0.010)	0.601 (0.020)	0.882 (0.016)	0.589 (0.021)	0.864 (0.017)	0.577 (0.024)	0.855 (0.019)
DTiGEMS+	0.853 (0.009)	**0.948 (0.007)**	**0.644 (0.018)**	0.934 (0.013)	0.631 (0.018)	0.916 (0.015)	0.618 (0.021)	0.904 (0.016)
NASNet-DTI	0.879 (0.008)	0.961 (0.006)	0.668 (0.017)	0.947 (0.012)	0.662 (0.017)	0.945 (0.012)	**0.646 (0.019)**	0.940 (0.014)
LNMGAT (ours)	0.873 (0.007)	0.958 (0.005)	0.671 (0.016)	0.952 (0.011)*	0.658 (0.015)	0.939 (0.011)	0.654 (0.017)	0.937 (0.013)

Color convention: best, 2nd-best, and **3rd-best**. Values are reported as mean with standard deviation in parentheses over 5-fold cross-validation. All baseline methods were reproduced in this study under the same preprocessing procedure, data partitions, cold-start scenario definitions, and pseudo-negative sampling protocol as LNMGAT., color convention indicates the best, second-best, and third-best values within each column. Statistical significance was assessed by comparing LNMGAT, with the strongest competing baseline in each setting using fold-level paired tests. * indicates p < 0.05, and ** indicates p < 0.01.

**TABLE 7:** Performance comparison of different methods under multiple scenarios on the BindingDB dataset.

Method	Warm start		Drug cold start		Target cold start		Pair cold start	
	AUPR	AUROC	AUPR	AUROC	AUPR	AUROC	AUPR	AUROC
MAARDTI	0.873 (0.009)	0.928 (0.006)	0.896 (0.011)	0.934 (0.008)	0.684 (0.018)	**0.938 (0.013)**	0.663 (0.020)	0.928 (0.014)
MGDTI	0.891 (0.008)	0.932 (0.005)	**0.902 (0.010)**	**0.939 (0.009)**	**0.689 (0.017)**	0.939 (0.012)	**0.666 (0.019)**	**0.933 (0.013)**
DrugLAMP	0.862 (0.010)	0.909 (0.008)	0.871 (0.012)	0.927 (0.009)	0.653 (0.019)	0.914 (0.014)	0.631 (0.021)	0.911 (0.015)
INDTI	0.878 (0.009)	0.921 (0.006)	0.889 (0.011)	0.948 (0.011)	0.668 (0.018)	0.924 (0.012)	0.647 (0.020)	0.922 (0.013)
MINDG	0.841 (0.012)	0.898 (0.010)	0.856 (0.014)	0.882 (0.017)	0.622 (0.021)	0.902 (0.015)	0.601 (0.023)	0.897 (0.017)
DTiGEMS+	0.884 (0.008)	0.926 (0.005)	0.897 (0.010)	0.934 (0.010)	0.682 (0.017)	0.931 (0.011)	0.659 (0.019)	0.928 (0.013)
NASNet-DTI	**0.889 (0.007)**	**0.931 (0.005)**	0.903 (0.010)	0.947 (0.009)	0.693 (0.016)	0.935 (0.010)	0.671 (0.018)	0.934 (0.012)
LNMGAT (ours)	0.923 (0.006)**	0.944 (0.004)**	0.916 (0.009)**	0.956 (0.009)*	0.705 (0.015)	0.947 (0.010)*	0.684 (0.017)**	0.941 (0.012)*

Color convention: best, 2nd-best, and **3rd-best**. Values are reported as mean with standard deviation in parentheses over 5-fold cross-validation. All baseline methods were reproduced in this study under the same preprocessing procedure, data partitions, cold-start scenario definitions, and pseudo-negative sampling protocol as LNMGAT., color convention indicates the best, second-best, and third-best values within each column. Statistical significance was assessed by comparing LNMGAT, with the strongest competing baseline in each setting using fold-level paired tests. * indicates p < 0.05, and ** indicates p < 0.01.

### 3.4 Baseline reproduction protocol and statistical analysis

To ensure a fair comparison, all baseline methods reported in Tables 4–7 were reproduced in this study under the same evaluation framework as LNMGAT. Specifically, LNMGAT and all baseline models were evaluated using the same dataset preprocessing procedure, binary label conversion strategy, 5-fold cross-validation partitions, cold-start scenario definitions, and pseudo-negative sampling protocol. For each dataset and each evaluation scenario, all methods were trained and tested on the same fold partitions.

The reported values are presented as mean and standard deviation across the five cross-validation folds. Therefore, the mean and standard deviation values in Tables 4–7 apply to both LNMGAT and all reproduced baseline methods. Each method was independently trained and evaluated on the five fold-specific training and test splits.

For statistical comparison, we compared LNMGAT with the strongest competing baseline in each evaluation setting using the fold-level performance values. Since all methods were evaluated on the same cross-validation folds, statistical significance was assessed using a fold-wise paired t-test. One asterisk indicates p < 0.05, and two asterisks indicate p < 0.01. Statistical markers are shown only when LNMGAT achieved the best performance and the improvement over the strongest competing baseline was statistically significant.

## 4 Results and validation

### 4.1 Overall performance of LNMGAT across benchmark datasets and evaluation settings

To comprehensively evaluate the effectiveness of our proposed LNMGAT model, we compared it with several representative methods developed in recent years, including MAARDTI (Zhan et al., 2025), MGDTI (He et al., 2025), DrugLAMP (Luo et al., 2024), INDTI (Chen et al., 2024), MINDG (Yang et al., 2024b), DTiGEMS+ (Thafar et al., 2020), and NASNet-DTI (Zhong and Du, 2025). The proposed LNMGAT model demonstrates consistently competitive performance across all evaluation settings on the Yamanishi, Davis, KIBA, and BindingDB benchmark datasets (Tables 4–7). Its effectiveness is observed in the warm-start setting and is more evident in the three cold-start protocols, which reflect practical DTI prediction scenarios involving unseen drugs, unseen targets, or novel drug–target pairs. LNMGAT obtains these results without the use of true negative samples during training, which indicates that the model can extract informative and generalizable representations under limited prior interaction evidence.

On the Yamanishi dataset (Table 4), LNMGAT achieves performance comparable to the strongest baselines in the warm-start setting (AUPR: 0.912 compared with 0.935 for MAARDTI; AUROC: 0.941 compared with 0.963 for MAARDTI). It obtains the highest scores in both the Drug Cold Start setting (AUPR: 0.565, AUROC: 0.603) and the Pair Cold Start setting (AUPR: 0.544, AUROC: 0.566). In the Target Cold Start setting, LNMGAT obtains an AUPR of 0.872, which is slightly lower than that of MAARDTI (0.875). A similar pattern is observed on the Davis dataset (Table 5). LNMGAT records the highest AUPR in the Drug Cold Start setting (0.648) and the Target Cold Start setting (0.662) and also records the highest AUROC values in these two settings (0.935 and 0.948). The model maintains competitive performance across the remaining evaluation conditions.

On the KIBA dataset (Table 6), which is larger and more diverse, LNMGAT maintains stable performance across all evaluation settings. It achieves the highest AUPR and AUROC in the Drug Cold Start setting (AUPR: 0.671, AUROC: 0.952). It also ranks among the top-performing methods in the Target Cold Start and Pair Cold Start settings. For example, in the Pair Cold Start setting, LNMGAT achieves an AUPR of 0.654, which is higher than the 0.646 obtained by NASNet-DTI. On the BindingDB dataset (Table 7), which contains heterogeneous protein families and chemical structures, LNMGAT achieves the highest performance in all evaluation settings. Its warm-start results reach an AUPR of 0.923 and an AUROC of 0.944. It also obtains the highest scores in the Drug Cold Start setting (AUPR: 0.916, AUROC: 0.956), the Target Cold Start setting (AUPR: 0.705, AUROC: 0.947), and the Pair Cold Start setting (AUPR: 0.684, AUROC: 0.941).

Across the four datasets, the four evaluation settings, and the two performance metrics (32 evaluation outcomes in total), LNMGAT exhibits stable performance. Among the 16 AUPR values, LNMGAT achieves the highest score in 10 cases and the second-highest score in 4 cases. Therefore, it ranks within the top two in 14 of the 16 AUPR outcomes. In the three cold-start settings, which consist of 12 AUPR values in total, LNMGAT achieves the highest performance in 9 cases and ranks second in the remaining 3 cases. These results indicate that LNMGAT has strong generalization ability when making predictions for unseen drugs, unseen targets, and novel drug–target pairs.

### 4.2 SwissDock molecular docking validation

To investigate the binding characteristics underlying our model’s predictions, we performed molecular docking using SwissDock (https://www.swissdock.ch) (Bugnon et al., 2024) based on the Yamanishi dataset. We chose the 5 highest (Top 5) and 5 lowest (Bottom 5) scoring drug-target pairs for docking validation.

**FIGURE 3:**
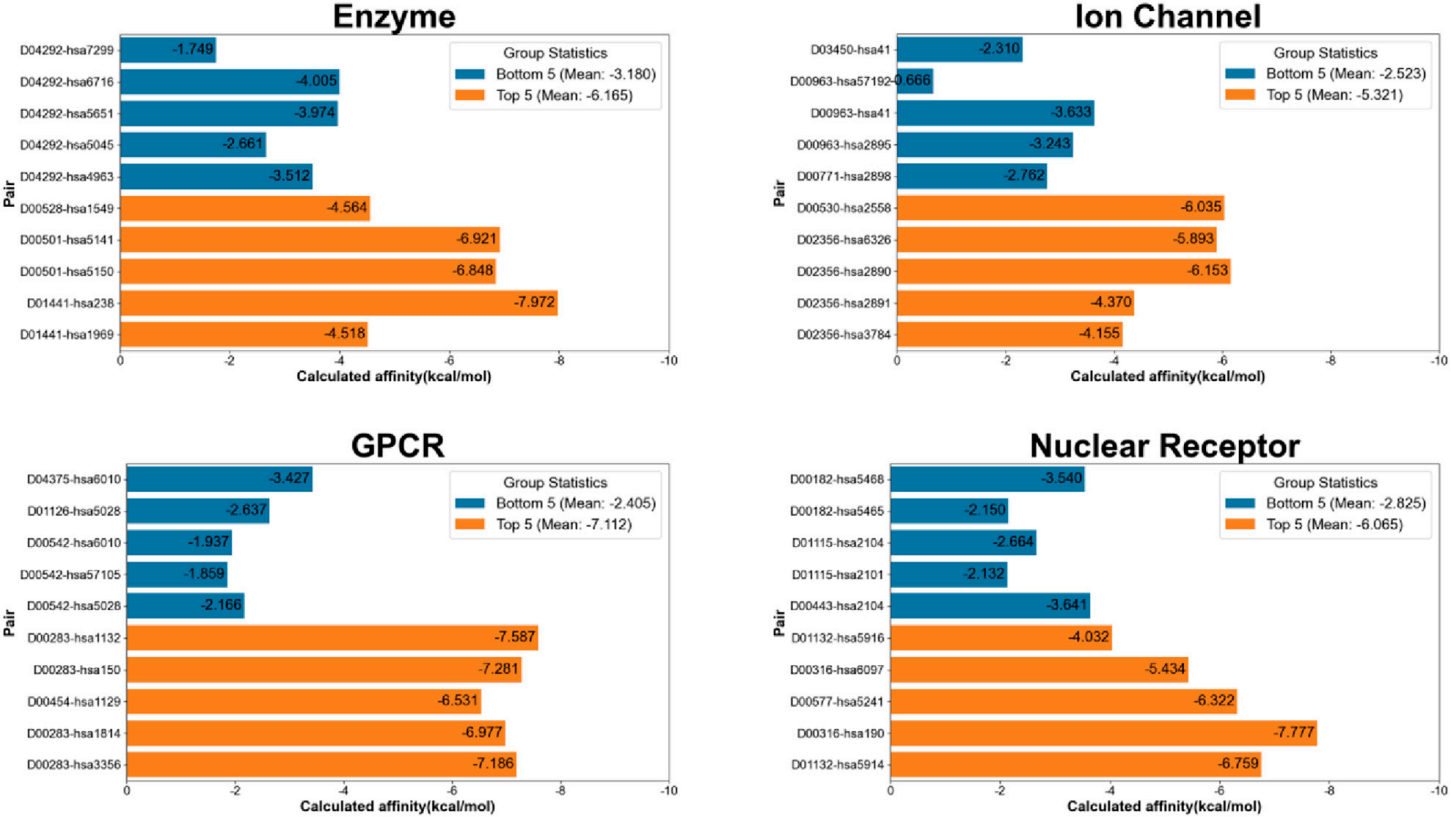
Quantitative evaluation of predicted drug–target binding affinities.

As illustrated in Figure 3, the comparative analysis reveals a clear separation in binding performance between the top five and bottom five drug–target pairs across all four protein classes. For Enzymes, the top-performing compounds show an average binding affinity of −6.165 kcal/mol, markedly stronger than the −3.180 kcal/mol observed for the lowest-ranked group. A similar trend is evident for Ion Channels (−5.321 vs. −2.523 kcal/mol) and Nuclear Receptors (−6.065 vs. −2.825 kcal/mol). The most pronounced difference appears in GPCRs, where the top five pairs achieve an average affinity of −7.112 kcal/mol, contrasting sharply with −2.405 kcal/mol for the bottom five.

Given that more negative binding affinity values indicate stronger and more energetically favorable interactions, these results demonstrate that the top-ranked drug–target pairs consistently form more stable complexes. This pattern underscores the predictive model’s ability to distinguish high-quality binders from weaker ones and highlights the potential of these top candidates for further experimental validation.

**FIGURE 4:**
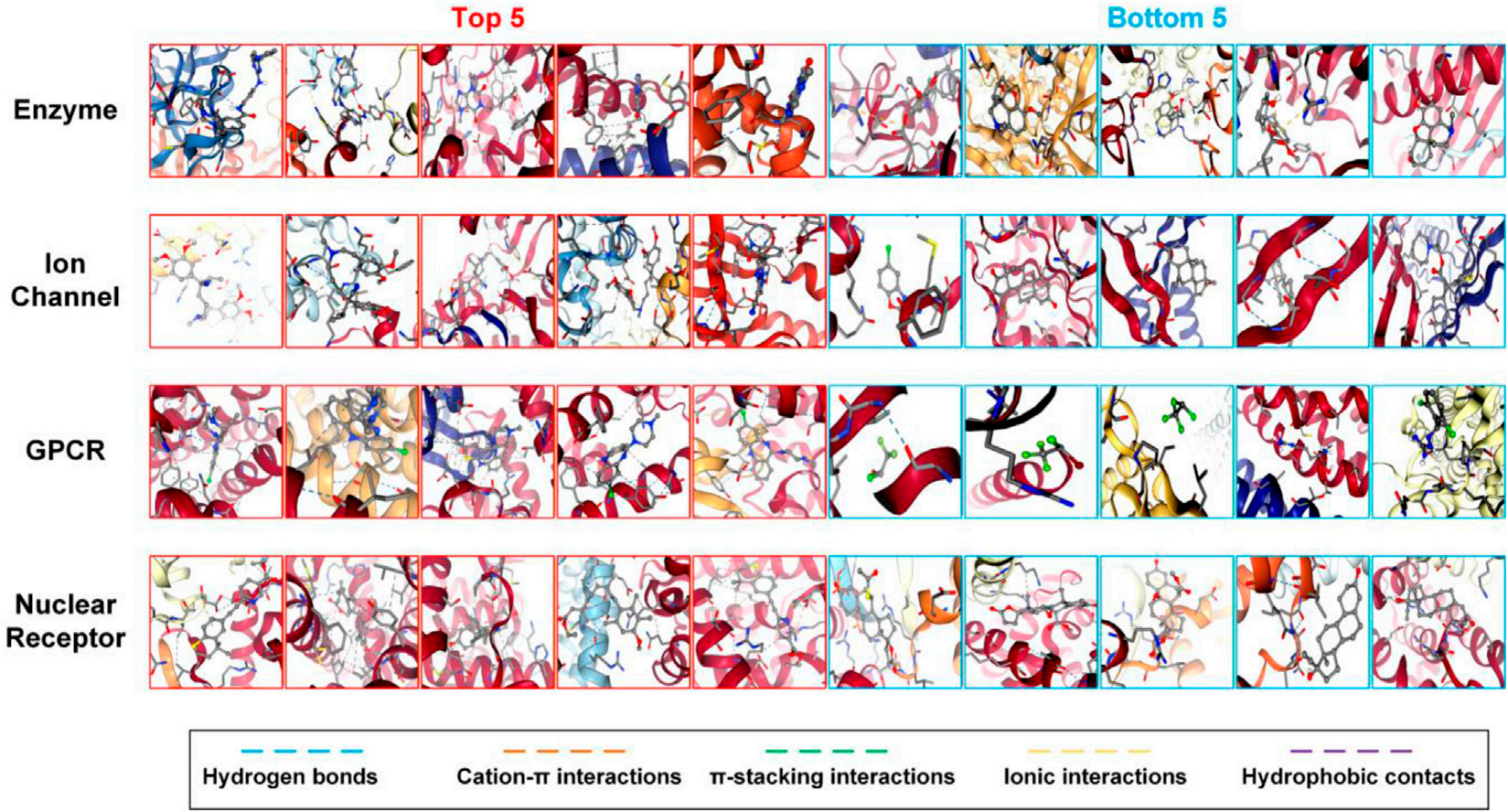
Visualization of molecular docking results.

**FIGURE 5:**
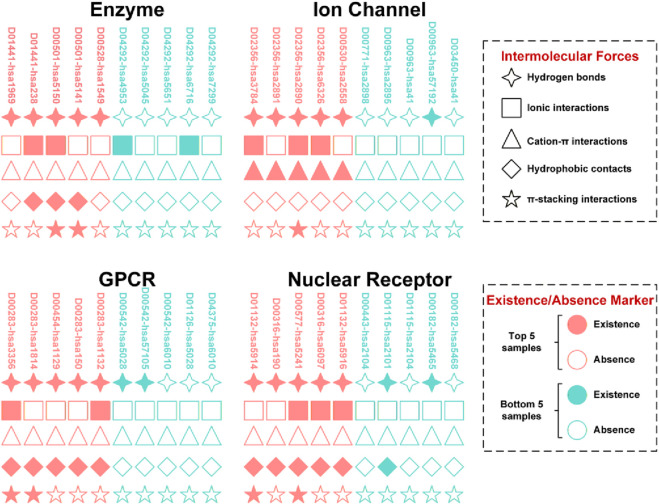
Statistics of intermolecular interaction types.

Furthermore, as shown in Figure 4, the top 5 pairs display markedly higher occurrences of key favorable non-covalent interactions compared with the bottom 5 pairs. The protein surface is shown in white, with ligands rendered as sticks. Key non-covalent interactions are indicated by colored dashed lines and surfaces: hydrogen bonds (cyan), cation-π interactions (orange), π–π stacking (green), ionic interactions (yellow), and hydrophobic contacts (purple). As clearly visible in the figure, the top-5 complexes (left half, red labels) consistently display abundant and well-organized non-covalent interactions. Multiple hydrogen bonds, aromatic stacking interactions, ionic or cation-π contacts, and extensive hydrophobic packing are simultaneously appear in most poses, resulting in ligands that are deeply buried in the respective binding pockets with highly complementary shapes and interaction patterns. In marked contrast, the bottom-5 complexes (right half, blue labels) exhibit dramatically fewer specific interactions. Many of these ligands make only one or two weak contacts, or are positioned on the protein surface with large portions exposed to solvent. Several poses show almost no recognizable favorable interactions at all, and the ligands frequently appear misplaced relative to the known functional sites. A clearer and more intuitive statistical summary of the intermolecular interactions is presented in Figure 5.

These quantitative and qualitative differences collectively demonstrate that high-scoring predictions from our model not only yield numerically superior binding affinities but also translate into richer and more diverse stabilizing molecular interactions. This strong agreement between predicted scores and actual docking outcomes validates the reliability and interpretability of the model for drug–target interaction prediction.

### 4.3 Independent validation on external datasets

To evaluate the model’s generalization capability on real-world data, we tested it on 43 experimentally validated drug–target interaction pairs collected from the DrugBank database, as shown in Table 8 (The SMILES of the compounds and the sequence information of the targets are provided in the Supplementary Table.). The model had been trained exclusively on the widely used Yamanishi benchmark dataset, and a strict cross-check confirmed that none of the DrugBank evaluation pairs—neither compounds nor protein targets—appeared in the training set. This ensured that the assessment was free from data leakage and genuinely reflected the model’s ability to generalize beyond the training distribution. Using a classification threshold of 0.5, where prediction scores ≥0.5 were considered positive interactions, the model correctly identified 35 of the 43 known DTI pairs, achieving an accuracy of 81.40%.

**TABLE 8:** Experimentally validated drug–target interaction pairs.

No.	Drug ID	Drug name	Target ID	Target name	Prediction
1	DB04272	Citric acid	P15121	Aldo-keto reductase family 1 member B1	0.9856
2	DB04272	Citric acid	Q9HBA0	Transient receptor potential cation channel subfamily V 4	0.7973
3	DB04272	Citric acid	O75390	Citrate synthase, mitochondrial	0.9994
4	DB00140	Riboflavin	Q969G6	Riboflavin kinase	0.2837
5	DB00477	Chlorpromazine	P14416	D (2) dopamine receptor	0.9850
6	DB03044	Doramapimod	P08631	Tyrosine-protein kinase HCK	0.9931
7	DB00788	Naproxen	P23219	Prostaglandin G/H synthase 1	0.8257
8	DB00795	Sulfasalazine	P09917	Polyunsaturated fatty acid 5-lipoxygenase	0.9564
9	DB00544	Fluorouracil	P04818	Thymidylate synthase	0.8287
10	DB00564	Carbamazepine	P43681	Neuronal acetylcholine receptor subunit alpha-4	0.7655
11	DB01159	Halothane	O14649	Potassium channel subfamily K member 3	0.7894
12	DB01159	Halothane	Q9NPC2	Potassium channel subfamily K member 9	0.9387
13	DB00818	Propofol	P47870	Gamma-aminobutyric acid receptor subunit beta-2	0.9679
14	DB00280	Disopyramide	Q14524	Sodium channel protein type 5 subunit alpha	0.2760
15	DB00280	Disopyramide	P11229	Muscarinic acetylcholine receptor M1	0.0014
16	DB00280	Disopyramide	P08172	Muscarinic acetylcholine receptor M2	0.6573
17	DB00312	Pentobarbital	P14867	Gamma-aminobutyric acid receptor subunit alpha-1	0.6868
18	DB00312	Pentobarbital	P47869	Gamma-aminobutyric acid receptor subunit alpha-2	0.9054
19	DB00514	Dextromethorphan	Q99720	Sigma non-opioid intracellular receptor 1	0.9770
20	DB00502	Haloperidol	P28335	5-Hydroxytryptamine receptor 2C	0.9891
21	DB00502	Haloperidol	P14416	D (2) dopamine receptor	0.9979
22	DB01235	Levodopa	P21728	D (1A) dopamine receptor	0.7964
23	DB01235	Levodopa	P21918	D (1B) dopamine receptor	0.7741
24	DB00770	Alprostadil	P34995	Prostaglandin E2 receptor EP1 subtype	0.9283
25	DB00708	Sufentanil	P35372	Mu-type opioid receptor	0.8580
26	DB00704	Naltrexone	P35372	Mu-type opioid receptor	0.0217
27	DB06594	Agomelatine	P28335	5-Hydroxytryptamine receptor 2C	0.8691
28	DB00315	Zolmitriptan	P28222	5-Hydroxytryptamine receptor 1B	0.7473
29	DB00315	Zolmitriptan	P28221	5-Hydroxytryptamine receptor 1D	0.8328
30	DB00315	Zolmitriptan	P30939	5-Hydroxytryptamine receptor 1F	0.8339
31	DB00627	Niacin	P49019	Hydroxycarboxylic acid receptor 3	0.3584
32	DB04540	Cholesterol	Q9Y5K3	Choline-phosphate cytidylyltransferase B	0.9232
33	DB04540	Cholesterol	Q14994	Nuclear receptor subfamily 1 group I member 3	0.4447
34	DB00255	Diethylstilbestrol	Q92731	Estrogen receptor beta	0.9345
35	DB06777	Chenodeoxycholic acid	Q96RI1	Bile acid receptor	0.8970
36	DB00367	Levonorgestrel	P06401	Progesterone receptor	0.2874
37	DB00367	Levonorgestrel	P18405	3-Oxo-5-alpha-steroid 4-dehydrogenase 1	0.5069
38	DB00255	Diethylstilbestrol	Q92731	Estrogen receptor beta	0.7438
39	DB01174	Phenobarbital	P14867	Gamma-aminobutyric acid receptor subunit alpha-1	0.9765
40	DB01039	Fenofibrate	Q07869	Peroxisome proliferator-activated receptor alpha	0.1973
41	DB00834	Mifepristone	P06401	Progesterone receptor	0.6430
42	DB02789	Pregnenolone	P11474	Steroid hormone receptor ERR1	0.9655
43	DB00636	Clofibrate	Q07869	Peroxisome proliferator-activated receptor alpha	0.7777

* The Drug ID, was obtained from the DrugBank database, while the Target ID, was sourced from the UniProt database. Color convention: Incorrect Predictions.

Most of the validated DTIs were assigned high prediction scores, indicating strong model confidence. Representative examples include Citric acid–Citrate synthase, mitochondrial (DB04272–O75390, 0.9994), Doramapimod–Tyrosine-protein kinase HCK (DB03044–P08631, 0.9931), Haloperidol–D (2) dopamine receptor (DB00502–P14416, 0.9979), and Phenobarbital–Gamma-aminobutyric acid receptor subunit alpha-1 (DB01174–P14867, 0.9765). These high-confidence predictions span diverse protein families—including enzymes, ion channels, GPCRs, and nuclear receptors—highlighting the model’s ability to capture a broad range of pharmacological interaction patterns.

Despite LNMGAT’s high performance across the majority of the externally validated interactions, several pairs of verified positive samples exhibited significantly low predicted scores, which constitutes the problem of False Negatives (FNs). For instance, the predicted score for the known positive pair Riboflavin–Riboflavin kinase (DB00140–Q969G6) was 0.2837, and for Disopyramide–Muscarinic receptor M1 (DB00280–P11229), the score was notably low at 0.0014. Since these pairs are confirmed interactions in the DrugBank database, these low scores represent clear prediction errors on the ground truth.

We hypothesize that these observed FNs stem from the inherent challenges posed by feature space coverage and training data distribution. Specifically, the structure of molecules like Riboflavin (a water-soluble vitamin) may possess unique structural characteristics that deviate significantly from the small-molecule drugs dominating the training corpus. This makes it challenging for the model to capture the subtle but critical interaction signatures. Similarly, the target proteins involved might possess sequences with low homology to the proteins frequently encountered during training, hindering the effectiveness of the sequence-based feature extractor and leading to a lack of sufficient information for accurate inductive prediction. Furthermore, LNMGAT’s training relies on large-scale DTI datasets where certain complex or non-typical interactions are inevitably underrepresented. The low-scoring, yet verified, positive pairs are likely edge cases that the model finds difficult to learn via simple similarity-based reasoning. It is crucial to remember that the model’s predicted score reflects the likelihood of interaction based on learned features, not an absolute measure of binding strength. These results underscore that despite the model’s general robustness, its performance is restricted when encountering drug-target pairs with sparse features or novel structural characteristics not well covered by the training distribution. In conclusion, the presence of these False Negatives highlights a primary area for future improvement. Future research should prioritize the integration of more powerful and expressive feature encoders capable of robustly representing structurally divergent molecules and targets. This will be essential to enhance LNMGAT’s generalization capacity and minimize predictive errors on non-typical, yet experimentally verified, interactions, especially in cold-start scenarios where features are inherently sparse.

### 4.4 Interpretability analysis

**FIGURE 6:**
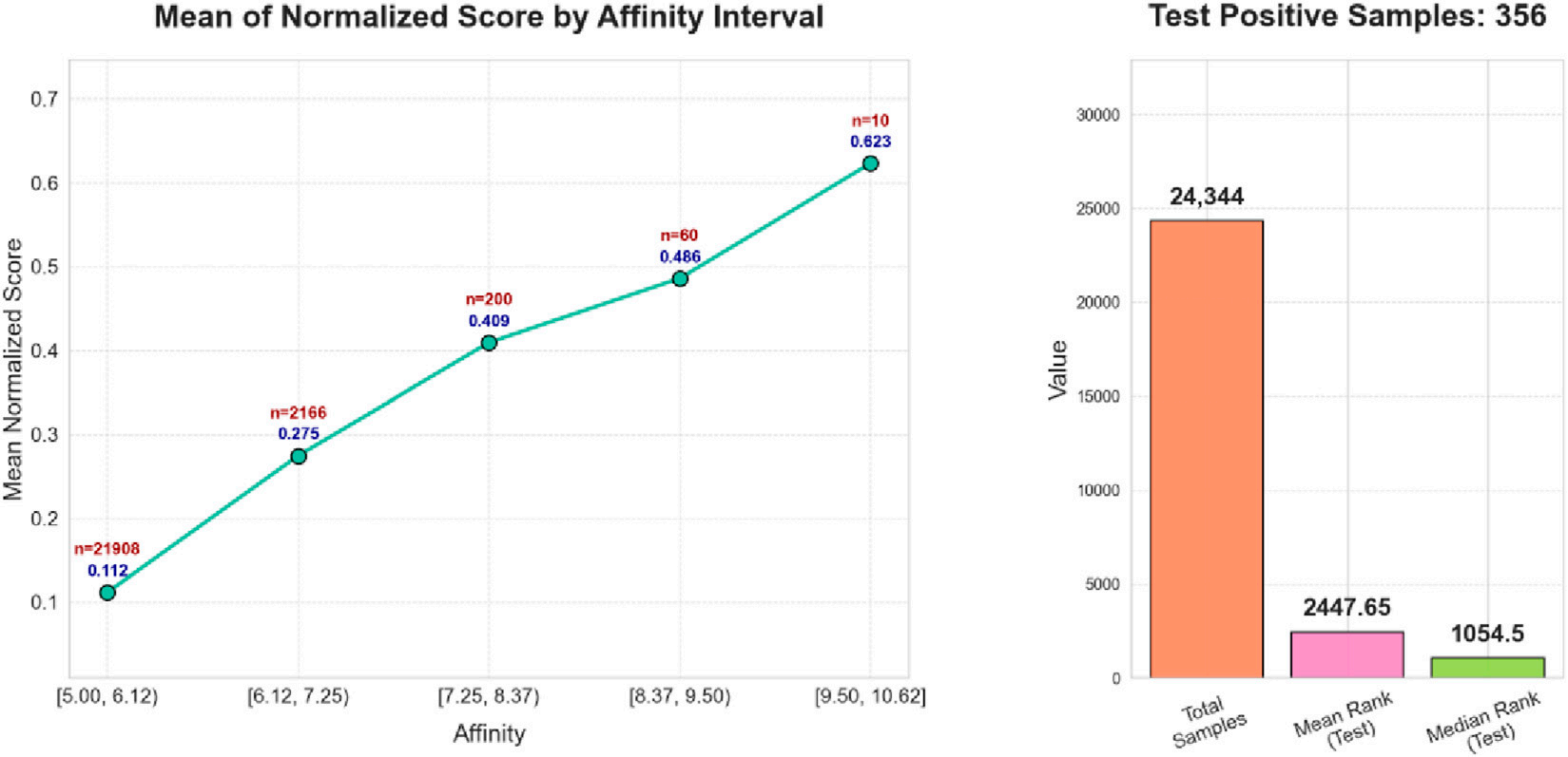
Between predicted scores and experimental affinity on the Davis datasets.

**FIGURE 7:**
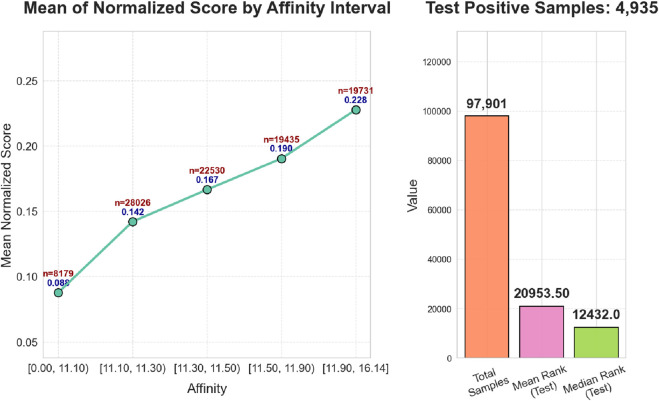
Between predicted scores and experimental affinity on the KIBA datasets.

To assess the interpretability of the LNMGAT model and evaluate the ability of its LapRLS module to capture real drug–target affinity patterns, we analyzed the relationship between predicted scores and experimental affinity values on both the Davis (Figure 6) and KIBA (Figure 7) datasets. For each dataset, 20% of the positive samples were randomly selected as the test set, and the predicted scores were grouped according to affinity intervals. In the line plots, the numerical annotations above each point denote the number of samples falling within the corresponding affinity interval as well as the mean normalized prediction score for that interval, allowing a direct comparison between sample density and prediction strength. In the Davis dataset, the mean normalized prediction scores exhibited a clear increasing trend as the affinity increased, rising from 0.112 in the lowest interval (5.00–6.12) to 0.623 in the highest interval (9.50–10.62). A similar yet more moderate upward trend was observed in the KIBA dataset, where the mean score increased from 0.088 in the lowest affinity interval (0.00–11.10) to 0.228 in the highest interval (11.90–16.14). These monotonic trends demonstrate that the LapRLS module effectively captures the underlying correspondence between similarity-based representations and true binding strength across datasets.

The ranking performance of test samples further reinforces this conclusion. In the Davis dataset, the test positives achieved a mean rank of 2447.65 and a median rank of 1054.5, both substantially better than what would be expected under random ordering. In the larger KIBA dataset, the mean and median ranks were 20,953.50 and 12,432.0, respectively, again indicating that the model consistently pushes true high-affinity interactions toward the top of the ranking list. This behavior reflects the model’s ability to distinguish strong binders from weak binders in realistic retrieval or screening scenarios.

Taken together, the consistent alignment between predicted scores and affinity intervals—along with the favorable ranking outcomes—provides strong evidence that the LapRLS component of LNMGAT effectively models real affinity relationships. These results confirm the robustness and biological interpretability of the model and underscore its practical relevance for supporting drug discovery applications such as virtual screening, candidate prioritization, and affinity-driven interaction analysis.

### 4.5 Hyperparameter sensitivity analysis

**FIGURE 8:**
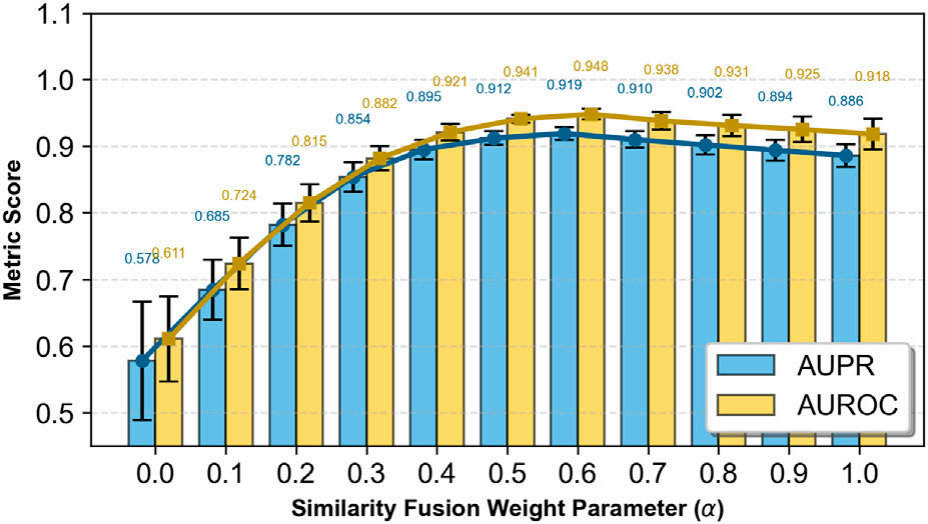
Sensitivity analysis of similarity matrix weights.

**FIGURE 9:**
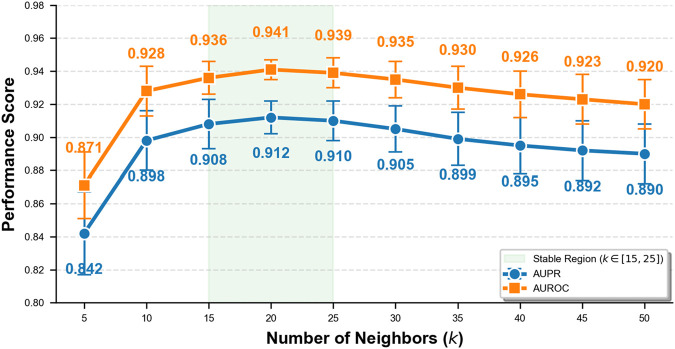
Sensitivity analysis of K value in KNN.

We further conduct a comprehensive hyperparameter sensitivity analysis to evaluate the robustness of the proposed framework with respect to two key hyperparameters: (1) the similarity fusion weight α in Equations 5, 2 the neighborhood size k used in the k-NN graph construction for drugs and targets. The results are reported in Figures 8, 9, respectively.

Figure 8 illustrates the impact of the similarity fusion weight α on model performance, evaluated on the Yamanishi dataset under the Warm Start setting. The parameter α controls the relative contribution of structural similarity and interaction-sharing similarity, and is varied from 0 to 1 with a step size of 0.1. As shown in Figure 8, when α=0 , the model relies solely on interaction-sharing similarity, resulting in substantially degraded performance (AUPR = 0.578, AUROC = 0.611). Conversely, when α=1 , only structural similarity is used, which also leads to a noticeable performance drop (AUPR = 0.886, AUROC = 0.918). These results indicate that either similarity source alone is insufficient to fully characterize drug--target interactions. In contrast, fusing the two similarity sources ( 0<α<1 ) consistently improves performance, demonstrating their strong complementarity. Notably, the model exhibits relatively stable performance across a wide range of α values (approximately $\alpha \in \left[0.3,0.9\right]$ ), indicating that the proposed method is not overly sensitive to the exact choice of the fusion weight. The best or near-optimal performance is achieved around α=0.5 , where the model attains an AUPR of 0.912 and an AUROC of 0.941 with small variance. This stability is particularly important for the LapRLS-based negative sample mining stage, as excessive bias toward either similarity source may introduce noisy or unreliable negative samples. The balanced fusion at α=0.5 therefore helps ensure robust negative sample selection and provides a stable supervision signal for subsequent graph-based representation learning.

Figure 9 presents the sensitivity analysis of the neighborhood size k used in constructing the k-NN graphs for drugs and targets. We vary k from 5 to 50 and evaluate the performance on the Yamanishi dataset under the Warm Start setting. When k is small (e.g., k=5 ), the constructed graphs are overly sparse, which limits information propagation across nodes and leads to suboptimal performance (AUPR = 0.842, AUROC = 0.871). As k increases, both AUPR and AUROC improve steadily, reaching their peak around k=20 (AUPR = 0.912, AUROC = 0.941). This suggests that a moderate neighborhood size is crucial for capturing sufficient local structural information while preserving meaningful graph topology. When k is further increased beyond 25, the performance gradually degrades. This decline can be attributed to the introduction of noisy or weakly relevant neighbors, which dilute discriminative structural signals and negatively affect representation learning. Importantly, the model maintains relatively stable performance within the range $k\in \left[15,25\right]$ , indicating that the proposed framework is robust to moderate variations of k .

Based on this analysis, we adopt α=0.5 and k=20 as the default hyperparameter settings in our experiments, as they provide a favorable balance between predictive accuracy, robustness, and computational efficiency.

### 4.6 Ablation study

To further examine the contribution of the two key components in LNMGAT, we conducted an ablation study on the Davis dataset using 5-fold cross-validation. Three model variants were compared: the full LNMGAT model, the LapRLS_Only variant, and the GAT_Only variant. The full LNMGAT integrates LapRLS-guided reliable pseudo-negative mining with dual multi-head GAT encoders. LapRLS_Only removes the GAT-based nonlinear representation learning module and performs prediction only through the similarity-propagation-based LapRLS model. GAT_Only retains the graph attention architecture but removes LapRLS-guided pseudo-negative preselection, thereby using random pseudo-negative sampling during training.

As shown in Table 9, the full LNMGAT consistently achieved the best AUPR across all four evaluation settings. In the warm-start setting, LNMGAT obtained an AUPR of 0.841, outperforming LapRLS_Only by 0.021 and GAT_Only by 0.167. This indicates that the dual GAT encoders can substantially improve representation learning beyond linear similarity propagation, while LapRLS-guided pseudo-negative mining provides more reliable supervision than random sampling. In the drug cold-start and target cold-start settings, LNMGAT also achieved the highest AUPR values of 0.648 and 0.662, respectively, showing that the integration of similarity-based pseudo-negative mining and graph attention learning improves generalization to unseen drugs or targets.

**TABLE 9:** Ablation study of LNMGAT on the Davis dataset under four evaluation settings.

Start type	Full LNMGAT	LapRLS only	GAT only
Warm start	0.841 (0.006)	0.820 (0.009)	0.674 (0.018)
Drug cold start	0.648 (0.019)	0.632 (0.012)	0.618 (0.011)
Target cold start	0.662 (0.015)	0.623 (0.010)	0.635 (0.013)
Pair cold start	0.624 (0.018)	0.500 (0.005)	0.598 (0.016)

Values are reported as mean AUPR, with standard deviation in parentheses over 5-fold cross-validation. Full LNMGAT, denotes the complete model with LapRLS-guided pseudo-negative mining and dual GAT, encoders. LapRLS_Only uses only the LapRLS-based similarity propagation module. GAT_Only retains the GAT, architecture but replaces LapRLS-guided pseudo-negative mining with random pseudo-negative sampling.

The advantage of the full model was most evident in the pair cold-start setting, where neither the drug nor the target appeared in the supervised training pairs. LNMGAT achieved an AUPR of 0.624, compared with 0.500 for LapRLS_Only and 0.598 for GAT_Only. The weak performance of LapRLS_Only in this setting suggests that linear similarity propagation alone is insufficient when both entities are unseen. Meanwhile, the performance gap between LNMGAT and GAT_Only indicates that reliable pseudo-negative mining remains beneficial even when nonlinear graph attention encoders are used. Overall, these results demonstrate that LapRLS and GAT play complementary roles: LapRLS improves the reliability of pseudo-negative supervision, whereas GAT enhances nonlinear entity representation learning. Their integration contributes to the stable performance of LNMGAT across both warm-start and cold-start prediction scenarios.

## 5 Discussion

One of the most significant contributions of this work is demonstrating that reliable and generalizable DTI prediction can be achieved without using any experimentally validated negative samples, a limitation that has long constrained the interpretability and biological fidelity of DTI models. Traditional frameworks generate negative examples by random sampling, an approach that implicitly assumes a vast majority of unknown pairs are true non-interactions. This assumption is often invalid and introduces substantial label noise, particularly in cold-start settings where little prior information is available. By contrast, LNMGAT incorporates a LapRLS-based negative mining module that exploits graph smoothness principles to infer reliable non-interacting pairs from drug–drug and target–target similarity structures. This mechanism establishes a biologically coherent supervisory signal and fundamentally reduces the risk of mislabeling potentially interacting pairs as negatives. Most importantly, a substantial body of research has demonstrated that LapRLS exhibits reliable performance across various interaction domains (Chen et al., 2016; Chen et al., 2012; Xia et al., 2010; Yang et al., 2025).

The elimination of dependence on true negative samples directly contributes to the model’s strong performance across all evaluation settings. LNMGAT consistently excels not only in warm-start tasks but also in the more challenging drug cold start, target cold start, and drug–target pair cold start scenarios. The model maintains high AUPR and AUROC values even when the test entities are entirely unseen during training, reflecting the effectiveness of its similarity-guided graph representation learning. By constructing and refining similarity-based k-NN graphs, the model captures transferable molecular and functional relationships that persist across different datasets. This capability is crucial in practical drug discovery workflows, where novel compounds or uncharacterized proteins frequently lack historical interaction data.

The results from the DrugBank external validation further underscore the benefits of our negative-sample–free design. The ability to correctly identify 35 out of 43 experimentally validated interactions without any overlap between training and testing drugs or targets illustrates the model’s strong generalization capacity and its avoidance of distribution-specific bias. Importantly, the cases with low prediction scores correspond to biochemically weak or context-dependent interactions, suggesting that LNMGAT does not overconfidently assign positive labels in ambiguous regions—a common failure mode of models trained with noisy random negatives.

Interpretability analysis reveals an additional layer of biological coherence. The monotonic alignment between predicted scores and affinity intervals indicates that the LapRLS-enhanced graph structure preserves the intrinsic topology of affinity-related signals. Furthermore, ranking analyses show that LNMGAT effectively prioritizes strong binders in large candidate sets, which mirrors the goals of virtual screening applications. The molecular docking experiments offer structural validation: pairs predicted as high-confidence interactions form more stable and energetically favorable binding complexes, supported by richer non-covalent interactions. This agreement across statistical, biochemical, and structural analyses provides supportive evidence that the model may capture biologically meaningful patterns beyond dataset-specific artifacts.

While LNMGAT addresses several core limitations of existing DTI methods, opportunities for future work remain. The similarity fusion strategy, while effective, relies on manually defined weights; incorporating adaptive or learnable similarity integration may further improve the quality of the constructed graphs. Additionally, enhancing similarity matrices using contrastive self-supervised learning could strengthen the LapRLS inference process. Incorporating 3D protein structures or ligand conformational ensembles may also benefit challenging cases involving flexible binding pockets. Lastly, further exploration of scalability will support deployment in real-world contexts involving very large chemical libraries or proteome-scale predictions.

The academic motivation of LNMGAT lies not only in improving benchmark performance, but also in addressing a common yet underemphasized limitation in DTI prediction: the uncertainty of negative labels. In many public DTI datasets, unobserved pairs are often treated as negative samples for model training, although they may include undiscovered interactions. This assumption can introduce label noise and affect the reliability of learned representations. By using LapRLS-guided reliable pseudo-negative mining, LNMGAT provides a practical strategy to reduce the dependence on randomly sampled negatives and to construct more reliable supervision from unlabeled data. Moreover, by evaluating the model under warm-start, drug cold-start, target cold-start, and pair cold-start settings, this study places more emphasis on generalization scenarios that are closer to real drug discovery applications. These aspects improve the methodological relevance and academic merit of the proposed framework.

Although LNMGAT achieved competitive performance across multiple benchmark datasets and cold-start settings, several aspects remain to be further improved. First, the pseudo-negative samples identified by LapRLS are inferred from unlabeled drug–target pairs and are not equivalent to experimentally verified non-interactions. Future studies should incorporate experimentally confirmed inactive or non-binding pairs when such data become available, thereby improving the reliability of negative supervision. Second, the current cold-start evaluation relies on fixed drug–drug and target–target similarity priors. More stringent fully inductive protocols, such as scaffold-based drug splitting and sequence-identity-based target splitting, should be explored to better assess generalization to truly novel compounds and targets. Third, the present external validation is mainly based on DrugBank records and molecular docking analysis. Future work should include target-matched active and inactive controls, prospective validation, and wet-lab binding assays for top-ranked candidate interactions. Finally, the representation capacity of LNMGAT could be further enhanced by integrating more expressive molecular and protein encoders, including multimodal drug representations, hybrid graph neural networks, and generative molecular modeling strategies (Dehghan et al., 2026). Recent studies on multimodal graph-based drug association prediction and low-data molecular generation provide useful directions for improving representation learning and cross-domain generalization in future DTI prediction frameworks (Gharaghani, 2026).

Overall, the discussion demonstrates that LNMGAT provides a principled and innovative framework that not only resolves the longstanding challenge of negative label uncertainty but also advances generalization, interpretability, and biological plausibility in DTI prediction. Its performance and design collectively position it as a valuable tool for modern computational drug discovery.

## 6 Conclusion

This study introduces LNMGAT, a negative-sample–free drug–target interaction prediction framework that integrates Laplacian regularization with graph attention–based representation learning. By replacing traditional random negative sampling with LapRLS-guided inference, LNMGAT effectively eliminates dependency on experimentally validated negative labels and constructs a more reliable, biologically grounded supervision signal. The model demonstrates strong and consistent performance across four benchmark datasets and excels in all cold-start scenarios, highlighting its suitability for predicting interactions involving novel drugs or previously uncharacterized targets. External validation on DrugBank further confirms its robust generalization ability, while interpretability and docking analyses verify the biological plausibility of its predictions. Collectively, these results suggest that LNMGAT is a competitive and potentially useful framework for virtual screening, drug repurposing, and large-scale pharmacological profiling. Future work will explore adaptive similarity learning and structural integration to further enhance the model’s transferability and applicability in real-world drug discovery.

## Data Availability

The datasets presented in this study can be found in online repositories. The names of the repository/repositories and accession number(s) can be found in the article/Supplementary Material.
